# Cold as a Therapeutic and Prophylactic Agent*Read at Monterey, Mexico, meeting of the International Medical Association, January 25, 1908.

**Published:** 1908-04

**Authors:** R. D. Robinson

**Affiliations:** Torreon, Coah., Mexico


For the Texas Medical Journal.

### Cold as a Therapeutic and Prophylactic Agent.*

BY R. D. ROBINSON, M. D., TORREON, COAH., MEXICO.

Cold, or the rapid extraction of heat by any form of refrigeration, is a remedy that can be relied upon in properly selected cases. The application of cold locally has two objects. First, to cause the contraction of local blood-vessels which, through the process of inflammation, have become so congested that the parts have become swollen and painful; second, to temporarily anesthetize nerve fibres for the immediate relief of pain. Pain and irritation bring more blood supply; more blood supply, more irritation and pain. Now, if cold will anesthetize the nerve fibers, there will be less pain and less irritation; less irritation, less blood supply; less blood supply, less irritation, etc.—purely a matter of mechanics and physics.

Thus, cold is a remedy for burns, bruises and sprains, or any injury which may be followed by inflammatory process. In some cases cold is not well tolerated when heat should be substituted. Such intolerance of cold is most likely to be found in neurasthenics.

From Bartholow's text-book I quote the following paragraph: "When a hand or foot is immersed in cold water, the temperature of the other extremity also falls. Cold abstracts the heat of the body, at least of its superficial surface, and affects the condition of the internal organs through the nervous system. It is through an influence transmitted from the peripheral distribution of the nerves of the hand to the center and thence reflected to corresponding anatomical nervous connections on the other side, that the of temperature is due when the other hand is immersed in cold water. We have a right to assume, therefore, that when cold water is applied to the whole surface of the body, changes of temperature take place within."

On entering a cold bath a sense of chilliness is experienced; the lips become blue, the pulse is quickened, the breath is gasping and the temperature of the surface is lowered by driving the blood supply from the surface into the great trunks. If the temperature of the bath is not too low reaction quickly takes place, the coldness and depression are succeeded by warmth and exhilaration, the pulse

*Read at Monterey, Mexico, meeting of the International Medical Association, January 25, 1908.

quickens and respiration becomes easy and unembarrassed, and general bodily vigor is increased which, in health, may be felt during the entire day. However, should the bath be continued too long, there is a corresponding depression. Coldness, weakened pulse and muscular weakness, followed by a sense of fatigue. "This is due to the prolonged extraction of heat and the accumulation of blood in the great trunks, thus interfering with metamorphosis of tissue." If these baths are properly given they act as a tonic; the circulation is invigorated, there is more rapid tissue change, the appetite is increased, the digestive organs are stimulated, the body will gain in weight, and the patient is less sensitive to temperature and climatic changes.

Any influence which will soothe and quiet the nervous symptoms of a fever patient and reduce temperature without reducing vitality, is greatly to be desired. The search for such a medium has occupied the minds of our greatest therapists for hundreds of years. It has been one of the principal questions which has occupied the best talent of our great manufacturing concerns, and has brought forward hundreds of preparations with all sorts of exaggerated claims.

No one even approached the solution of the problem until the busy doctor, by reason of necessity, and in the face of tremendous prejudice, began to study the value of hydrotherapy and the application of cold as a remedy.

These observations were exchanged in the sick room, in the medical societies and in the medical press, until now the entire medical profession of the world is giving much valuable time to its study. It is the only means by which temperature may be reduced, the nervous system soothed and quieted and by the same process stimulate the vital forces.

Modern medicine has profited more from this source than any other, unless serum therapy could claim first place. This, however, I am not yet willing to admit, as the use of serum thus far is quite limited, while that of cold has the widest possible range. (This statement does not include the application of asepsis and antisepsis in surgery.)

It is commonly stated by most authorities that heat and cold are equal in their physiological effects and that the choice of the patient may be relied upon as to which should be used. This is entirely wrong. I ask the pardon of this association for thus disputing the entire body of our eminent authorities, but cold is entirely superior and should always have the preference, except in a few selected cases, such as supersensitive patients or where pus formation is suspected. Cold contracts muscular fiber and hardens tissue and might thus force pus to seek an outlet in the wrong direction, and for its power to contract muscular fiber, might increase pain.

Cold may be applied in many ways. The cold wet pack may be made by wringing a couple of sheets out in ice water and wrapping the entire body in it, then cover closely with several blankets.

The time in the bath may be from fifteen minutes to an hour, depending on the object in view. If stimulation or invigoration or lowering of temperature is desired, fifteen to twenty minutes is sufficient. For lowering temperature, the bath may be repeated every hour to every four hours as the urgency of the case may require. If diaphorisis is desired it will require an hour. Diaphorisis may be aided by taking a glass of ice water during the bath.

A cold compress for local applications may be made with an ice bag, ice water cap, or towels wrung out in ice water and applied over the affected part.

In cases such as pneumonia the entire thorax should be wrapped in large thick compresses. These compresses should be wrung out in ice water at least every hour. Some suggest every five minutes, but this is entirely too often, as such frequent changing molests the patient beyond tolerance. I have found it better to change about every hour and keep one or two ice bags bound over the affected parts. In peritonitis, acute appendicitis, ilio-colitis, typhoid or any acute intestinal congestion or inflammation, ice bags may be bound over the affected part. The same is true of cystitis, metritis, nephritis, hepatitis, meningitis or any acute inflammation or congestion of any part. It is best not to apply an ice bag, not having a cloth covering, next the skin.

Tub baths, shower baths and sponging are means of applying cold to the entire body. Another effective means of rapid refrigeration is sponging the body with alcohol, following with vigorous fanning. This is especially valuable in supersensitive patients.

In tonsilitis, diphtheria and croup an ice compress at the throat and pieces of ice held in the mouth are palliating and extremely serviceable. Spasmodic croup is usually quickly and safely relieved by cold compress at the throat, while cold sponging will almost certainly ward off an oncoming attack.

The use of cold baths, douches, compresses, packs, etc., in the treatment of fevers is too well known to be taken up at this time. I wish to insist, however, that the use of cold and cold water in fevers is of the most vital importance; indeed, it is our most valuable asset. As previously stated, this is the only remedy that will reduce temperature and at the same time stimulate vital forces.

In acute conjunctivitis, mastoiditis or any acute inflammatory action of the eye, ear, nose or throat, the cold compress is the remedy "par excellence."

In hyperpyrexia the cold bath is our only safe recourse. Before the use of cold was well understood, a temperature of 108 degrees F. was considered fatal. Now, in acute rheumatism, delirium tremens, malaria and thermal fever, a temperature of 112 degrees F. has been controlled and brought safely down by the prompt use of the cold bath.

Fevers such as typhoid, malaria, scarlatina, measles, smallpox, and cerebro-spinal meningitis are advantageously treated with the cold wet pack; especially when temperature is high with delirium, stupor and scanty urine.

Inflammatory affections of the meninges, meningeal hemorrhage, cerebral hemorrhage, hematemesis, hemoptysis, hematuria, injuries to the spinal cord and meninges, thermal fever, delirium tremens, infantile convulsions, pleuritis, pneumonia and inflammatory affections within the chest, are conditions favorably influenced by the application of cold. Don't give up a case of sciatica until you have tried the application of cold. Bind several ice bags along the track of the nerve and keep them there night and day. You will often relieve sciatica in this way when all other means have failed.

Neuralgia of the supra-orbital nerve may often be entirely relieved by freezing the skin directly over the seat of the nerve. For this purpose a piece of ice covered with a little fine salt and firmly pressed over the nerve, or a tube of ethyl chloride sprayed on the skin along the seat of the nerve, serves admirably.

The cold, wet dressing for various injuries, lacerations and contusions, is so important I wish to call especial attention to this feature. Most injuries of this kind are covered with dirt, grease and all kinds of filth, and usually occur several hours or several days away from the surgeon. It wet dressings are applied at once and kept wet with coldest water obtainable, the wound may be kept reasonably aseptic for many hours. Wounds thus treated are rarely swollen or painful. The constant application of cold water will bewilder the most persistent microbe in existence and will often put him entirely out of business.

As an illustration of cases extending over a period of several years, I will mention a hand badly crushed and lacerated in a railway accident. The wound had all kinds of dirt and filth ground into the tissues. I had the wound irrigated for three hours in clean cold well water, sterilized water could not be obtained. I then carefully trimmed and freshened the wounds and secured union by first intention. Not a drop of pus followed. Cold, wet dressings were applied for the first twenty-four hours, then dry dressings. In badly infected wounds cold, wet dressings should be alternated with dry dressings to prevent too much hardening of tissues. In treatment of wounds by submersion, hot water at about 105 degrees F. should be used.

A piece of ice bound snugly to the anus is a prompt and effective means of reducing prolapsis or acute external hemorrhoids. It relieves the pain, contracts the blood-vessels and drives the blood away from tumors. The irritation being thus relieved, there is a rapid return to the normal condition. A piece of ice inserted into the rectum acts in the same manner on internal hemorrhoids, and is a means of relief in cystitis and prostatitis, while an ice suppository will provoke the morning stool. Enemas of ice water for the morning evacuation of the bowel are superior to warm water, from the fact that enemas of warm water require one to two quarts, while those of ice water require two to four ounces. Ice water enemas are also beneficial in internal or bleeding piles.

A tumbler full of ice water on arising acts as a laxative; its action is increased by the addition of a teaspoonful of salt. Hot water will do as well, but is nauseating to most patients.

A piece of ice applied directly to the skin is an effectual local anesthetic; under its influence minor incisions and punctures may be made without pain.

Frequently infarction or capillary embolism may be dispersed by the vigorous application of cold, thus many times avoiding dangerous abscesses. This should be watched closely, however, and discontinued as soon as pus has formed. In abscesses after pus has been withdrawn and the cavity treated, cold is the best application. It contracts the distended blood-vessels, relieves the pain and hastens resolution. An ice bag applied over the seat of a painful surgical operation acts like magic.

Strangulated hernia may often be reduced after having been packed in ice for an hour or more. Non-suppurating bubos or suppurating bubos after pus has been removed, orchitis, epididymitis, and such inflammations, are relieved and recovery hastened by prompt and constant use of ice compresses. Uterine hemorrhage may often be quickly arrested by inserting a piece of ice or an ice water douche.

In endocarditis and pericarditis the application of an ice bag over the heart relieves palpitation and pain, diminishes inflammation and quiets the heart.

In the disease of infant nutrition, known as summer complaint, gastro-intestinal fever, entero-colitis and various other names, there is no one thing a physician can do that will give quicker results than ice to the head and a cold compress over the entire abdomen. It quiets the nervous symptoms, lowers temperature, slows the pulse and produces sleep.

"Colds," which are prevalent from Pole to Pole and at all seasons, are most quickly and safely relieved by the application of cold. Give a cold bath two to four times a day. It may be a tub, shower or sponge bath and as cold as can be tolerated. Two to five minutes in the bath is ample and should be followed by brisk rubbing until reaction is established. A cold compress over the chest in case there is bronchial congestion or cough. The cough will cease and in an hour the patient will be asleep. After one becomes heated from exercise or working in a close warm room, a quick sponge bath in cold water is almost certain protection from muscular soreness or "taking cold."

If there ever was anything in the theory that "like cures like," it is in the application of cold for cold. What Northern boy has not frozen his nose, ears, hands or feet and thawed them out in a snowbank and not suffered any of the after consequences? What Northern boy has not, on mornings when the thermometer registered 10 to 20 degrees below, jumped out of bed, taken a snow bath and gone downtown without a topcoat, and scorned the idea that it was a cold day? Such boys are unusually free from "colds." As a prophylaxis against "colds" or pulmonary affections, the morning cold bath is king, queen and the whole royal family.

One of the most general and important uses of cold is the application of the morning cold bath to increase the general tone of the system. In cases of nervous exhaustion, loss of vivacity and vitality, and in anemia and chlorosis, improvement is certain and in many cases complete recovery results. The bath may be begun in water at 70 to 80 degrees F. and reduced daily until a temperature of 50 or 40 degrees F. is reached. The bath should be quick, lasting not more than two or three minutes and followed by brisk rubbing until reaction is established. It is claimed that many people can not tolerate these baths, but I have not found it so. The only cases I have found to whom this treatment is inapplicable are those who are too indolent to take the morning sponge bath. These cases are hopeless, and all the tonics in Christendom will do them no good.

The use of the morning cold bath as a systemic tonic and invigorant, is based on the following phenomena which are known to accompany a cold bath. As authority for these phenomena, I quote the following paragraph from Hare: "On entering the water he shivers, thinks it almost unbearably cold, his teeth chatter and he gasps if the cold suddenly touches the belly-wall or an equally sensitive surface. In a moment, however, reaction sets in, and the extremities, heretofore blue and trembling, become warmer and flushed. The pulse is increased in force and frequency, and the respirations are deeper and more thoroughly performed. As a result of this each portion of the body receives a more perfect supply of blood and feels rejuvenated. Following this stage of exhilaration a third comes on, in which the blueness and depression of the first stage recur in an exaggerated degree, but this condition does not ensue unless the person remains too long in the water. If he leaves the bath while in the acme of his exhilaration the stimulus may remain with him throughout the rest of the day.

"The reason for the occurrence of this train of symptoms is not far to seek. The chilliness of the first stage shows that the great abstraction of heat is lowering the bodily temperature; the centers for calorification in the body are not manufacturing all the heat that is needed for the preservation of the normal temperature. At first the cold drives the blood hurrying into the warm recesses of the body, leaving the surface of the body cold; but in a few moments the system is aroused to the recognition of the fact that it must increase its exertions in the propulsion of blood and manufacture of heat, and so, with an effort it puts forth all its power, picks up each corpuscle that is hiding from the cold in the internal organs, and after imbuing it with warmth obtained by increased heat production in the sources of heat manufacture, forces it out to the surface of the body along with its fellows, which are driven to all parts of the system. This is not a mere figurative way of putting the matter, for cold always contracts blood-vessels and reflexly stimulates the vital centers to increased activity."

The moral effect of the morning bath is not to be overlooked, and to emphasize this feature, I will offer the following quotation:

"A man who bathes every day, 9 to 1 he changes his linens every day. He not only feels better but he feels nearer to God; the moral effect is worth the trouble. The man who bathes each day will sin less than he who does not; he walks out from his home in the morning and feels that he is a clean man and that he has no desire to enter into the trickery of the common herd."

